# Einfluss von Richtmikrofonie auf die Höranstrengung bei Mittelohrimplantatträgern

**DOI:** 10.1007/s00106-022-01223-4

**Published:** 2022-09-26

**Authors:** Daniela Hollfelder, Lukas Prein, Tim Jürgens, Anke Leichtle, Karl-Ludwig Bruchhage

**Affiliations:** 1 Klinik für Hals- Nasen- und Ohrenheilkunde, Universitätsklinikum Schleswig-Holstein, Ratzeburger Allee 160, 23562 Lübeck, Deutschland; 2 Institut für Akustik, Technische Hochschule Lübeck, Mönkhofer Weg 239, 23562 Lübeck, Deutschland

**Keywords:** Hörbelastung, Vibrant Soundbridge, Mikrofontechnologie, Binaurales Hören, Räumliches Hören, Schwerhörigkeit, Burden of listening, Vibrant soundbridge, Microphone technology, Binaural hearing, Spatial hearing, Hearing loss

## Abstract

**Hintergrund und Ziel der Arbeit:**

Neben dem Sprachverstehen ist die empfundene Höranstrengung im Alltag ein wichtiger Indikator für den Erfolg einer Hörhilfe und deren Signalverarbeitung. Diese Studie hat zum Ziel, die subjektive Höranstrengung für Sprache im Störgeräusch bei Patienten mit dem aktiven Mittelohrimplantat Vibrant Soundbridge (VSB) für omnidirektionale und direktionale Richtwirkung und für einseitiges und beidseitiges Hören zu ermitteln.

**Material und Methoden:**

Bei 15 VSB-Trägern wurde die subjektive Höranstrengung in einem Lautsprecherring im reflexionsarmen Raum mit der adaptiven Skalierungsmethode ACALES (Adaptive CAtegorical Listening Effort Scaling) erfasst. Verschiedene Hintergrund-Störgeräusche aus unterschiedlichen Richtungen und gleichzeitig dargebotene Sätze aus dem Oldenburger Satztest wurden zu vier alltagsnahen akustischen Szenen kombiniert.

**Ergebnisse:**

Direktionale Richtwirkung verringerte die Höranstrengung bei VSB-Trägern im Median nur numerisch, insbesondere bei räumlich verteilten Störquellen und niedrigem Signal-Rausch-Verhältnis, allerdings ohne signifikanten Unterschied zur omnidirektionalen Einstellung. Einseitiges Hören mit VSB (bei Verschluss des kontralateralen Ohrs) führte dazu, dass im Probandenmittel alle untersuchten Höranstrengungskategorien bei signifikant höheren Signal-Rausch-Verhältnissen gemessen wurden als bei beidseitigem Hören.

**Schlussfolgerung:**

Insgesamt konnte keine statistisch signifikant niedrigere Höranstrengung bei Verwendung des Richtmikrofonprogramms nachgewiesen werden, daher liefert diese Studie keine Empfehlung für oder gegen das Richtmikrofonprogramm. Allerdings konnte eine geringere Höranstrengung bei beidseitigem Hören im Gegensatz zu einseitigem Hören gezeigt werden. Patienten sollten daher ermutigt werden, immer mit beiden Ohren bestmöglich versorgt zu hören.

## Abstract (en)

**Background and objective:**

Besides speech intelligibility, subjective listening effort is an important outcome for the success of hearing devices and their signal processing. The aim of the present study was to determine subjective listening effort for speech in a noisy background in patients with the active middle ear implant Vibrant Soundbridge (VSB) for omnidirectional and directional microphone settings, with and without occlusion of the contralateral ear.

**Material and methods:**

A total of 15 patients using a VSB were measured using the adaptive categorical listening effort scaling (ACALES) method in a ring of loudspeakers placed in an anechoic room. Different background noises from different directions and simultaneously presented sentences from the Oldenburg sentence test were combined in four different realistic acoustic scenes.

**Results:**

The directional microphone program reduced median subjective listening effort only numerically compared to the omnidirectional microphone program in acoustic scenarios with diffuse noise and with low signal-to-noise ratios; however, this difference failed to reach statistical significance. When occluding the ear contralateral to the VSB, all investigated listening effort categories were measured at significantly higher signal-to-noise ratios than with access to both ears.

**Conclusion:**

Due to missing statistical significance in reduction of listening effort, this study delivered no recommendation for or against usage of the directional microphone program; however, reduced listening effort was shown for binaural listening in comparison to monaural listening. Therefore, patients should be encouraged to always listen with both ears for best results.

Die Fähigkeit der Kommunikation in akustisch komplexen Situationen bedarf einer leistungsfähigen auditorischen Verarbeitung. Für Normalhörende spielt insbesondere das Hören mit zwei Ohren (beidseitiges Hören) eine große Rolle, um in räumlichen Situationen mit verteilten Störgeräuschquellen Sprache zu verstehen [5]. Schwerhörigkeit erschwert die Kommunikationsfähigkeit in solchen Situationen (z. B. in einer Cafeteria oder einem Restaurant) und kann zu einer größeren Anstrengung führen, dem Gespräch folgen zu können [17].

Einem schwerhörigen Patienten stehen in Abhängigkeit von der zugrunde liegenden Pathologie und dem Grad der Schwerhörigkeit verschiedene operative und technische hörverbessernde Maßnahmen zur Verfügung [6, 9]. Eine Möglichkeit bietet das aktive transkutane Mittelohrimplantat Vibrant Soundbridge (VSB) des Herstellers Med-El (Innsbruck, Österreich). Der Indikationsbereich für eine VSB [4] besteht für Patienten ab dem 5. Lebensjahr mit nichtprogredienter Schwerhörigkeit, einem maximalen Innenohrhörverlust von 65 dB HL im Hochtonbereich [9] und bestehender Kontraindikation für eine Hörgeräteversorgung. Die Hörgeräte-Kontraindikation kann entweder gegeben sein bei fehlendem Gehörgang und/oder Ohrmuschel [8] und/oder chronischer Otorrhö, bei chronischer Otitis media, rezidivierender Otitis externa und/oder Vorliegen einer Radikalhöhle, die den Verschluss des Gehörgangs durch ein externes Hörsystem oder ein Maßohrstück nicht möglich macht [6, 9]. Der Diskriminationsverlust bei maximalem Verstehen im Freiburger Einsilbertest sollte bei Vorliegen einer mittel- bis hochgradigen Innenohrschwerhörigkeit nicht mehr als 40 % betragen, da der Übergang zur Versorgung mit einem Cochleaimplantat (CI) fließend ist und in Erwägung gezogen werden muss.

Die VSB besteht aus zwei Komponenten: dem extern getragenen Audioprozessor (Samba oder Samba 2) und einem Implantat („vibrating ossicular prosthesis“, VORP 503), das aktiv die mechanische Bewegung der Mittelohrstrukturen verstärkt und damit den Hörverlust ausgleichen kann. Deshalb spricht man von einem aktiven Mittelohrimplantat. Das VORP 503 besteht einerseits aus der Empfängerspule, auf welche die durch den Audioprozessor vorverarbeitete Information elektromagnetisch übertragen wird. Der nachgeschaltete Demodulator dekodiert die Informationen und leitet sie an das aktive Bewegungselement, den FMT („floating mass transducer“), weiter. Der FMT wird vom Chirurgen mit einem entsprechenden Kuppler (Tab. 1) je nach Pathologie des Mittelohrs und entsprechender Indikation an Strukturen des Mittelohrs (Amboss, Steigbügel) oder am runden Fenster positioniert, um die verstärkte Bewegungsenergie optimal auf das Innenohr zu übertragen.

**Table 1:** 

Patienten-ID	Alter (Jahre)	Geschlecht	Seite	Pathologie	Anzahl Voroperationen	Tragedauer (Monate)	Versorgung kontralateral	Kopplung
ID 1	58	W	L	OS	3	39	NH	SP
ID 2	44	W	R	CH	2	1	HG	ST
ID 3	60	M	R	GGSt	2	12	HG	SP
ID 4	64	W	L	CH	1	22	HG	RW
ID 5	66	W	R	CH	2	17	HG	ST
ID 6	61	M	R	CH	3	21	HG	ST
ID 7	20	W	L	CH	4	12	NH	SP
ID 8	55	W	R L	CH CH	1 1	8 6	VSB VSB	ST RW
ID 9	43	M	R	OE	0	10	BB	SP
ID 10	48	W	R L	CH CH	1 2	42 29	VSB VSB	ST ST
ID 11	57	M	R	CH	2	86	NH	ST
ID 12	35	M	L	CH	3	2	NH	RW
ID 13	24	W	R	CH	1	96	NH	ST
ID 14	34	M	R	CH	3	1	NH	RW
ID 15	50	M	L	CH	2	6	NH	RW

*ID* Kennung, *L* links, *R* rechts, *OS* Otosklerose, *CH* Cholesteatom, *GGSt* Gehörgangsstenose, *OE* Otitis externa, *NH* normalhörend, *HG* Hörgerät, *VSB* Vibrant Soundbridge, *BB* Bonebridge, *SP* Amboss, kurzer Fortsatz, *ST* Stapes, *RW* „roundwindow“, rundes Fenster

Der extern getragene, mittels Magnetkraft gehaltene Audioprozessor überträgt das digital kodierte, verstärkte akustische Signal transkutan durch die intakte Haut auf die subkutan platzierte Spule des VORP 503. Die Erstaktivierung erfolgt nach der Einheilungsphase in etwa 4 Wochen nach dem operativen Eingriff.

Im extern getragenen Audioprozessor wird neben der omnidirektionalen Einstellung ein Programm mit adaptiver Richtmikrofontechnologie (direktional) angeboten. Die Richtcharakteristik des aus der Hörgerätetechnik bekannten adaptiven Richtmikrofons hat den Vorteil, dass Hintergrundgeräusche, die nicht in dem frontalen Sichtfeld des Endverbrauchers liegen, deutlich reduziert werden können [6, 16]. Dadurch konnte gezeigt werden, dass sich die Sprachverständlichkeit für bilaterale Hörgeräteträger [14, 20] und für bilaterale [2] und bimodale [25] Cochleaimplantatträger in räumlichen Umgebungen mit Störgeräusch signifikant verbessert.

In der audiologischen Forschung bekommt das Thema Höranstrengung, also die Messung des Aufwands während der Kommunikation in definierten Hörsituationen, einen großen Stellenwert [10]. Höranstrengung wird als „eine spezifische Form der geistigen Anstrengung [definiert], die auftritt, wenn eine Aufgabe das Zuhören beinhaltet“ (übersetzt aus [17]). Erste systematische Untersuchungen dazu wurden in den frühen 2000er-Jahren bei Normalhörenden (NH) durchgeführt [10, 15]. Die Ermittlung der Höranstrengung kann auf drei verschiedene Arten erfolgen [10, 15]:

- mittels physiologischer (objektiver) Messverfahren (z. B. Pupillometrie, Hautleitwertsmessung, Veränderung der Hirnaktivität) oder
- mittels kognitiver Leistungsverfahren (z. B. in einem Dual-Task-Experiment, um die selektive Aufmerksamkeit zu erfassen) oder
- mittels subjektiver Testmethoden (z. B. Fragebögen/kategoriale Höranstrengungsermittlung).

Das Ziel dieser Studie ist die Untersuchung der Höranstrengung von VSB-Patienten in alltagsnahen, räumlichen, störgeräuschbehafteten Situationen mit einer Methode, der eine subjektive Bewertung des Probanden zugrunde liegt. Ermittelt wurde die Höranstrengung mit dem adaptiven Höranstrengungs-Skalierungsverfahren (Adaptive Categorical Listening Effort Scaling; ACALES, [12]). Für diese Studie wurden folgende Forschungsfragen formuliert:

1. In welchen Hörsituationen bewirkt das Richtmikrofonsystem eine Veränderung der Höranstrengung?
2. Haben VSB-Patienten bei Verwendung beider Ohren eine geringere Höranstrengung als bei Verwendung nur ihres implantierten Ohrs mit Okklusion des Gegenohrs?

## Studiendesign und Untersuchungsmethoden

### Patienten

An der Studie nahmen 15 Patienten im Alter zwischen 20 und 66 Jahren (Ø: 48 Jahre, 7 männlich, 8 weiblich) teil. Die Tab. 1 bietet eine Übersicht über Pathologie, Tragedauer der VSB und Kopplungsart. Die postoperativen unversorgten Knochenleitungs- (KL) und Luftleitungsschwellen (LL) sind als Durchschnitt der vier Frequenzen im Hauptsprachbereich (PTA4) in Tab. 2 dargestellt und zeigen ipsilateral durchweg eine mittel- bis hochgradig kombinierte Schwerhörigkeit. Audiologisches Kriterium für den Studieneinschluss war ein Einsilberverstehen (ESV) mit VSB bei 65 dB SPL von $\geq$ 70 % bei Okklusion des Gegenohrs. Der Zeitraum zwischen VSB-Erstaktivierung und Messungen dieser Studie betrug im Durchschnitt 24 Monate.

**Table 2:** 

	Unversorgt				ESV (Freiburger im FF mit VSB; %)	
Pat.-ID	PTA4_KL_ (dB HL) ipsilateral	PTA4_LL_ (dB HL) ipsilateral	PTA4_KL_ (dB HL) kontralateral	PTA4_LL_ (dB HL) kontralateral	65 dB SPL in Ruhe ipsilateral	65 dB SPL im SS (60 dB SPL) ipsilateral
ID 1	37,5	61,25	17,5	21,25	95	55
ID 2	46,25	57,5	38,75	41,25	90	25
ID 3	31,25	52,5	37,5	53,75	95	55
ID 4	38,75	91,25	25	45	50	25
ID 5^a^	25	43,75	40	50	90	50
ID 6^a^	18,75	50	25	53,75	80	55
ID 7	12,5	56,25	5	6,25	100	60
ID 8	21,25 31,25	58,75 66,25	31,25 21,25	66,25 58,75	95 90	30 50
ID 9	15	47,5	13,75	46,25	100	60
ID 10	31,25 38,75	40 50	38,75 31,25	50 40	95 90	30 30
ID 11	23,75	58,75	2,5	3,75	90	40
ID 12	22,5	51,25	10	12,5	90	75
ID 13	11,25	46,25	8,75	10	95	60
ID 14	23,75	81,25	17,5	20	100	60
ID 15^a^	16,25	52,5	10	21,25	85	70

Tonaudiometrische Daten (PTA4: Mittelwert 0,5; 1; 2 und 4 kHz) der Knochenleitungs- (PTA4_KL_) und Luftleitungsschwellen (PTA4_LL_) des mit Vibrant Soundbridge (VSB) versorgten (ipsilateral) und des kontralateralen Ohrs, Einsilberverstehen (ESV) im Freifeld (FF) in Ruhe und mit Störschall (SS)

^a^Patient, dessen Datensatz nicht ausgewertet werden konnte

Um möglichst viele Patienten in die Studie einschließen zu können, wurden große interindividuelle Unterschiede der Hörfähigkeit und Versorgung kontralateral zugelassen (s. kontralaterale PTA4 in Tab. 2). Dazu wurden sowohl bilateral (*n* = 2), bimodal (*n* = 6) und unilateral (*n* = 7) mit VSB versorgte Probanden eingeschlossen.

Die bimodale Versorgungsform umfasste in dieser Studie (Tab. 1) auf der kontralateralen Seite sowohl Hörgeräte (*n* = 5) als auch das aktive Knochenleitungsimplantat Bonebridge (*n* = 1) des Herstellers Med-El. Unilateral bedeutet in diesem Zusammenhang, dass das gegenüberliegende Ohr normalhörend war.

Alle Probanden gaben ihr schriftliches Einverständnis zur Durchführung der Studie und bekamen eine Aufwandsentschädigung. Die Studie wurde begutachtet und befürwortet als im Einklang mit nationalem Recht und mit der Deklaration von Helsinki (1975) stehend durch die medizinische Ethikkommission des Universitätsklinikums Schleswig-Holstein, Standort Lübeck (AZ 20-019).

### Messverfahren – Bewertungsskala und Pegelsteuerung

Beim Messverfahren ACALES [11] sind die Probanden aufgefordert, subjektiv einzuschätzen, wie anstrengend es in der jeweiligen Situation ist, dem Zielsprachmaterial zu folgen. Als Zielsprachmaterial dienen dabei die Sätze des Oldenburger Satztestes (OLSA [21]), wobei das Sprachverstehen selbst nicht gemessen wird. Die Aufgabenstellung wird durch Auswahl einer der 14 Kategorien der Bewertungsskala beantwortet [12]. Diese Bewertungsskala besteht aus acht beschrifteten Kategorien („mühelos“ bis „extrem anstrengend“ und „nur Störgeräusch“) und sechs unbeschrifteten Zwischenschritten (Tab. 3). Jeder Wahlmöglichkeit wird ein numerischer Wert zugeordnet („effort scale categorial units“, ESCU). Die Kategorie „nur Störgeräusch“ kann ausgewählt werden, wenn der/die Proband(in) keine Sprachsignale, sondern lediglich das Störgeräusch wahrnimmt [11, 12].

**Table 3:** 

Kategorie	ESCU
Mühelos	1
Sehr wenig anstrengend	3
Wenig anstrengend	5
Mittelgradig anstrengend	7
Deutlich anstrengend	9
Sehr anstrengend	11
Extrem anstrengend	13
Nur Störgeräusch	14

*ESCU* „effort scale categorial units“

Während der Durchführung wurde der Pegel des Störgeräusches (60 dB A) konstant gehalten und der Pegel des Zielsprachmaterials, und somit auch das Signal-Rausch-Verhältnis (SNR) adaptiv verändert. Die genaue Pegelsteuerung ist hierbei in [3, 12] beschrieben. Der maximale Darbietungspegel des Sprachmaterials wurde für diese Studie auf 90 dB (A) limitiert. Das Start-SNR wurde auf 0 dB (entspricht 60 dB (A) Sprache und 60 dB (A) Störgeräusch) festgelegt, um sicherzustellen, dass bei der ersten Darbietung nicht ausschließlich Störgeräusch gehört wird, aber auch die Situation nicht komplett mühelos eingeordnet wird.

### Messaufbau und Kalibrierung

Die Studie wurde im reflexionsarmen Raum der Technischen Hochschule Lübeck durchgeführt. Der Proband saß auf einem Stuhl in der Mitte eines aus acht Lautsprechern (GENELEC 8020A Studio Monitor, Iisalmi, Finnland) bestehenden Rings mit gleichen Abständen zwischen benachbarten Lautsprechern. Der Abstand zwischen Proband und den auf Kopfhöhe eingestellten Lautsprechern (1,10 m vom Boden) betrug 1,00 m, dabei wurde keine Fixierung der Kopfpositionierung durchgeführt. Die Testsignale wurden auf einem PC mit MATLAB erzeugt (MATLAB R2018b, The Mathworks, Natick, USA) und über eine externe Soundkarte (RME Fireface UFX+, Version 1.63, Software Version 42, Haimhausen, Deutschland) an die Lautsprecher ausgegeben. Um diese Lautsprecher anzusteuern, wurde die Audio-Schnittstelle mSOUND in MATLAB verwendet [7]. Die Wahl der Höranstrengungskategorie erfolgte über ein Tablet. Der Versuchsleiter saß während des Versuchs im selben reflexionsarmen Raum. Eine Hi-Pro 2 USB (Natus Medical, San Carlos, USA), sowie das entsprechende Kabel zur Ansteuerung des Audioprozessors (CS64 inkl. Battery Pill 675) befand sich ebenfalls im Raum, um den Programmwechsel zwischen den einzelnen Konditionen vornehmen zu können.

Das jeweilige Testsignal wurde auf einen Schalldruckpegel von 60 dB (A) im Zentrum des Lautsprecherkreises (Kopfposition) kalibriert (Pegelmesser: Brüel & Kjær Typ 2250 mit Mikrofon Typ 4192, Nærum, Dänemark). Jeder Lautsprecher und jedes Störgeräusch (OL-Noise, Cafeteria-Noise, Babble-Noise) wurden dabei einzeln kalibriert. Die Darbietung erfolgte ebenfalls über die externe Soundkarte, in MATLAB wurden individuelle Korrekturwerte für die Lautsprecher berücksichtigt und programmiert.

### Stimuli und akustische Szenen

Als Sprachsignal wurden Sätze des Oldenburger-Satztests (OLSA) [21] verwendet. Das Sprachsignal wurde in allen Experimenten aus dem frontalen (0°) Lautsprecher dargeboten. Mittels des Lautsprecherrings und drei unterschiedlichen Störgeräuschen wurden insgesamt vier unterschiedliche akustische Szenarios generiert:

- (1) S0°N0°: Das stationäre OL-Noise [21] wurde über denselben frontalen Lautsprecher dargeboten wie das Sprachsignal. Diese Situation ist dieselbe wie in [12] und wurde zur Ermittlung der Höranstrengung bei stationärem Hintergrundgeräusch verwendet.
- (2) S0°NCafe: Ein Cafeteria-Noise (CAN aus [2]) wurde zeitgleich zum Sprachsignal über die fünf Lautsprecher der hinteren Hemisphäre (90°, 135°, 180°, 225°, 270°) präsentiert. Das CAN enthält sowohl kurze Dialog-Passagen zwischen zwei Sprechern, diffuse Hintergrundsprache und typische Cafeteria-Geräusche wie z. B. Geschirr- und Besteck-Geklapper. Die Lautsprechersignale waren dabei zeitlich unkorreliert. Hiermit soll eine alltagsähnliche Restaurant-Situation simuliert werden.
- (3) S0°N180°: Das Babble-Noise (aus [2] und [25]) wurde zeitgleich zum Sprachsignal über den Lautsprecher hinter dem Probanden (180°) dargeboten. Es handelt sich hierbei um die Überlagerung von 20 verschiedenen Sprechern zu einem Signal. Durch Wahl des Lautsprechers hinter dem Probanden wird die größtmögliche räumliche Trennung von Sprache und Störsignal erreicht.
- (4) S0°Nräumlich: Das Babble-Noise (aus [2] und [25]) wurde zeitgleich zum Sprachsignal über die fünf Lautsprecher (untereinander zeitlich unkorreliert) der hinteren Hemisphäre (90°, 135°, 180°, 225°, 270°) präsentiert, um eine große Gesprächsrunde zu simulieren.

Das Signal der einzelnen Störgeräusch-Lautsprecher wurde in den Szenen S0°NCafe und S0°Nräumlich jeweils um 7 dB abgeschwächt, damit der Gesamtpegel im Zentrum des Kreises bei gleichzeitiger Darbietung über die fünf Lautsprecher der gleiche war wie bei Verwendung nur eines Lautsprechers für das Störgeräusch (60 dB A).

In jedem der vier akustischen Szenarios wurde sowohl mit omnidirektionalem als auch mit direktionalem Programm gemessen (Übersicht in Tab. 4) ohne Okklusion des Gegenohrs.

**Table 4:** 

Nr.	Training/Test	Mikrofon – Charakteristik	Störgeräusch	Akustisches Szenario	Einseitig/beidseitig
1	Training	Omnidirektional	OL-Noise	S0°N0°	Beidseitig
2	Test	Omnidirektional	OL-Noise	S0°N0°	Beidseitig
3	Test	Direktional	OL-Noise	S0°N0°	Beidseitig
4	Test	Omnidirektional	Cafeteria-Noise	S0°NCafe	Beidseitig
5	Test	Direktional	Cafeteria-Noise	S0°NCafe	Beidseitig
6	Test	Omnidirektional	Babble-Noise	S0°N180°	Beidseitig
7	Test	Direktional	Babble-Noise	S0°N180°	Beidseitig
8	Test	Omnidirektional	Babble-Noise	S0°Nräumlich	Beidseitig
9	Test	Direktional	Babble-Noise	S0°Nräumlich	Beidseitig
10	Test	Omnidirektional	Babble-Noise	S0°Nräumlich	Einseitig
11	Test	Direktional	Babble-Noise	S0°Nräumlich	Einseitig

Um isoliert das VSB versorgte Ohr zu testen, wurde in der Situation S0°Nräumlich das Gegenohr mit Gehörschutz okkludiert und zusätzlich mit zirkumauralen Kopfhörer abgedämpft (Tab. 4, Nr. 10 + 11).

Jede Darbietung bestand aus drei randomisiert ausgewählten OLSA-Sätzen, die mit Pausen von 400 ms nacheinander dargeboten wurden. Das Sprachsignal war damit insgesamt 7,4 s lang. Das experimentspezifische Störgeräusch hatte 200 ms Vorlauf und war kontinuierlich so lange zu hören (durch eine knackfreie Dauerschleife), bis der Proband eine Höranstrengungskategorie ausgewählt hatte. Um Artefakte zu vermeiden, wurde der Sprache-Rauschen-Mix zu Beginn mit 50 ms An- und Abstiegsflanken versehen. Die Tab. 4 bietet eine Übersicht aller getesteten Konditionen.

### Messablauf

Die Verstärkungseinstellungen der Probanden wurden auf einen Studienprozessor kopiert. Im Rahmen dieser Studie wurde ausschließlich der Samba-Hi-Prozessor verwendet, der bei Neuimplantationen seit 07/2020 vom Nachfolgemodell Samba 2 Hi abgelöst wurde. Es wurden alle Zusatz-Features (Sprach- und Störlärm-Manager, Sound Smoothing, Windgeräusch-Unterdrückung und Feedback-Stopper) deaktiviert. Ein Programm 1 (omnidirektional) und Programm 2 (direktional) mit identischen Verstärkungswerten wurden programmiert. Umschalttöne wurden deaktiviert, um diese Programme gegenüber dem Probanden zu verblinden. Es folgte ein Funktionstest (Vibrogramm) des VORP 503 und ein Ton- und Sprachaudiogramm mit dem Freiburger Einsilbertest mit 20er-Wörterlisten.

Die Probanden bewerteten über ein Tablet (Lenovo Yoga) die Höranstrengung für das Zuhören des frontalen Sprechers in der jeweiligen Kondition (Tab. 4). Diese Konditionen wurden automatisiert und in vollständig randomisierter Reihenfolge ausgegeben. Eine dieser durchschnittlichen Testkonditionen dauerte etwa zwei bis drei Minuten mit anschließender Pause von zwei Minuten. Nach der Hälfte aller möglichen Konditionen wurde eine Pause von sieben Minuten ermöglicht.

### Verwendete Analyse und Statistik

Nach jeder Höranstrengungsskalierung wurde eine Regressionskurve mit zwei Geradenabschnitten, die durch einen runden Übergangsbereich verbunden sind, durch die erfassten Höranstrengungsdaten gemäß Vorschlag von [12] erzeugt. Diese Regressionskurve stellt die individuelle Höranstrengungsleistung dar. Um die Darstellung der mittleren Höranstrengung je Kondition und Probandengruppe zu erstellen, wurden die aufgenommenen Daten (ESCU-Wert und SNR) über die Probanden innerhalb einer Probandengruppe gepoolt. Eine mittlere Regressionskurve wurde durch Mittelung der individuellen SNR-Werte für jeden ganzzahligen ESCU-Wert erstellt. Werte der Kategorie „nur Störgeräusch“, wurden wegen Deckeneffekten bei der Erstellung der jeweiligen Regressionskurve nicht berücksichtigt.

Zur weiteren statistischen Auswertung wurde aus jeder individuellen Regressionskurve jedes Probanden der L1-Wert, d. h. der SNR zu Kategorie 1 („mühelos“), L7-Wert zu Kategorie 7 („moderat“) und L13-Wert zu Kategorie 13 („extrem anstrengend“) ermittelt. Die individuellen L1-, L7- und L13-Werte wurden anschließend mit MATLAB (R2018b) als Boxplots dargestellt. Zum Test auf Normalverteilung der Daten wurde der Shapiro-Wilk-Test verwendet. Hierbei wurde jede Messkondition einzeln überprüft. Dies ergab, dass mehr als 10 % der Konditionen nicht normalverteilt waren. Daher wurde jeweils der nichtparametrische Friedman-Test mit Messwiederholung und post hoc der Wilcoxon Rangsummentest verwendet. Die gesamte statistische Auswertung der Ergebnisse wurden mit der Software Jamovi (Version 1.6.4.0) bei einem Signifikanzniveau von 5 % durchgeführt.

## Ergebnisse

Es konnten nur 12 Datensätze in die Auswertung mit einfließen, da drei Probanden (Nr. 5, 6 und 15) je mehr als zehn Mal geringe Höranstrengungswerte (z. B. 3 ESCU) bei SNR < −25 dB angegeben haben. Bei solch niedrigen SNR sind gemäß der normativen Daten aus [12] selbst bei Normalhörenden Höranstrengungswerte von mindestens 10 ESCU zu erwarten, da das Sprachsignal nicht mehr verstanden wird. Dies legt nahe, dass die Probanden Nr. 5, 6 und 15 die Aufgabenstellung nicht korrekt verstanden haben.

### Richtmikrofonwirkung bei beidseitigem Hören

**Figure 1:**
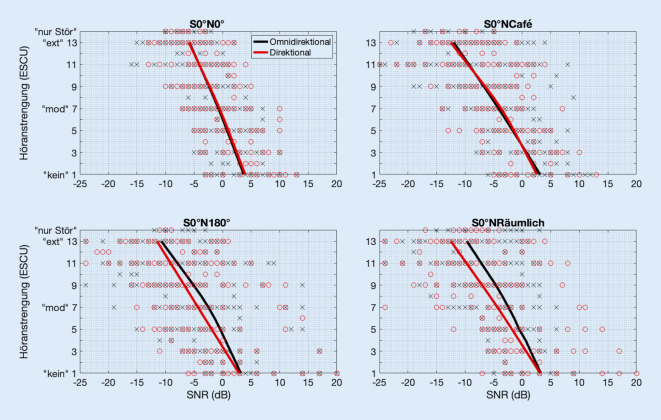

Die Abb. 1 zeigt die Höranstrengungswerte der zwölf VSB-Träger in den vier akustischen Szenarien als einzelne Diagramme, wobei in jedem Diagramm sowohl die Werte mit dem omnidirektionalen Programm (blaue Kreuze) als auch die Werte mit dem direktionalen Programm (rote Kreise) enthalten sind. Die mittlere Regressionskurve wurde für jedes akustische Szenario separat für omnidirektionales und direktionales Programm berechnet aus den individuellen Regressionskurven und jeweils als blaue (omnidirektional) bzw. rote (direktional) Linie in das Diagramm eingetragen. Es zeigt sich eine hohe Streuung der Rohdaten (Kreise und Kreuze) mit etwas kleinerem Ausmaß in der Kondition S0°N0°. Die Regressionskurven liegen in jedem Diagramm dicht beieinander, außer in den Diagrammen S0°N180° und S0°Nräumlich gibt es bei niedrigen SNR bzw. hohen Höranstrengungswerten eine leichte Abweichung. Hier ist die rote Regressionskurve zu niedrigeren SNR versetzt.

**Figure 2:**
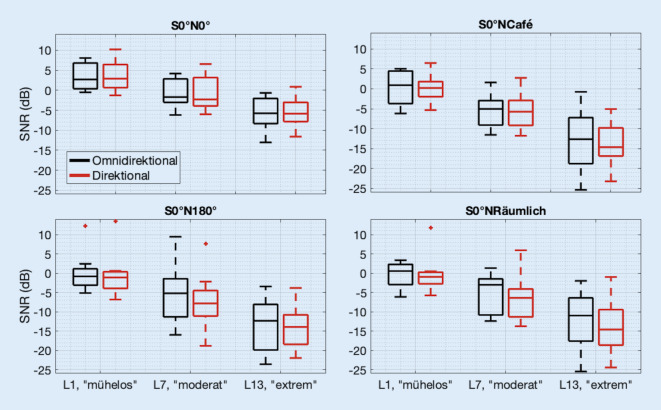

Für die weitere statistische Auswertung wurden aus den individuellen Regressionskurven jeweils der L1-Wert, L7-Wert und L13-Wert extrahiert. Die Verteilung dieser Höranstrengungskategorie-Werte ist als Boxplot in Abb. 2 dargestellt. Blaue Boxplots stellen die Daten in der omnidirektionalen Einstellung und rote Boxplots die Daten in der direktionalen Einstellung dar. Oberes und unteres Ende der Box zeigen jeweils das 25. und 75. Perzentil der Daten, und der horizontale Strich in der Box ist der Median. Whiskers erstrecken sich bis auf das 1,5fache der Länge zwischen Box-Ende und Median. Wie aus Abb. 1 aufgrund der großen Streuung der Rohdaten zu erwarten, zeigt sich ein großer Überlapp der Interquartilbereiche zwischen den beiden Programmen.

Der Friedman-Test ergab für S0°N0° eine signifikante Abhängigkeit von der Höranstrengungskategorie (χ^2^ = 24,0; *p* < 0,001), aber keine Abhängigkeit vom Hörprogramm für die L1-Werte (χ^2^ = 0,333; *p* = 0,564), die L7-Werte (χ^2^ = 3,00; *p* = 0,083) und die L13-Werte (χ^2^ = 3,00; *p* = 0,083). Für S0°NCafe ergab sich ebenfalls eine signifikante Abhängigkeit von der Höranstrengungskategorie (χ^2^ = 24,0; *p* < 0,001), aber keine Abhängigkeit vom Hörprogramm für die L1-Werte (χ^2^ = 0,333; *p* = 0,564), die L7-Werte (χ^2^ = 1,33; *p* = 0,248) und die L13-Werte (χ^2^ = 0,00; *p* = 1,00). Der Friedman-Test ergab für S0°N180° eine signifikante Abhängigkeit von der Höranstrengungskategorie (χ^2^ = 24,0; *p* < 0,001), aber keine Abhängigkeit vom Hörprogramm für die L1-Werte (χ^2^ = 0,333; *p* = 0,564), die L7-Werte (χ^2^ = 0,00; *p* = 1,00) und die L13-Werte (χ^2^ = 0,00; *p* = 1,00). Für S0°NRäumlich ergab sich ebenfalls eine signifikante Abhängigkeit von der Höranstrengungskategorie (χ^2^ = 24,0; *p* < 0,001), keine Abhängigkeit vom Hörprogramm für die L1-Werte (χ^2^ = 0,333; *p* = 0,564), L7-Werte (χ^2^ = 1,33; *p* = 0,248), L13-Werte (χ^2^ = 1,33; *p* = 0,248).

Die Statistik unterstützt somit die Nullhypothese, dass in dieser Studie für beidohriges Hören keine Höranstrengungsunterschiede zwischen den Hörprogrammen gefunden wurden. Allerdings zeigen die Regressionskurven leicht niedrigere SNR bei gleicher Höranstrengung für das direktionale Programm, insbesondere für die Kondition S0°NRäumlich bei mittleren und hohen Höranstrengungskategorien (hier: L7-Werte und L13-Werte), also insbesondere in den Situationen, die die Probanden als besonders schwierig empfanden. Die Differenz der L7-Mediane beträgt zwischen den beiden Programmen 3,4 dB, die Differenz der L13-Mediane beträgt hier 3,6 dB, die Differenz der L1-Mediane aber nur 1,5 dB.

### Vergleich von Höranstrengung bei beidseitigem und einseitigem Hören

**Figure 3:**
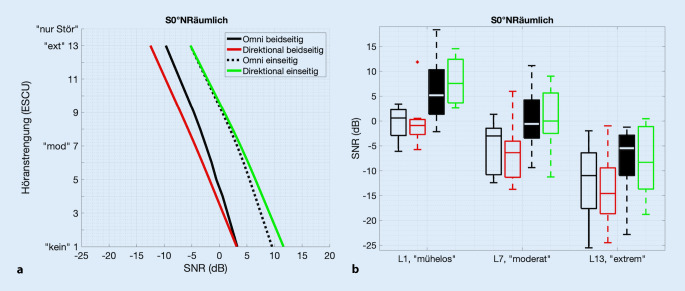

Die Abb. 3 oben zeigt die gemittelten Höranstrengungskurven für das akustische Szenario S0°NRäumlich sowohl für beidseitiges Hören (blau und rot) als auch für einseitiges Hören. Das einseitige Hören wurde realisiert durch Verschluss der Gegenseite (schwarz und grün), also Abnahme jeglicher Hörhilfe auf der Gegenseite zusätzlich zu einem Ohrstöpsel und halbseitigem Aufsetzen von zirkumauralem Gehörschutz. Zu beachten ist hier, dass die beiden bilateral mit VSB versorgten Probanden hier nur mit ihrer linken Seite in die Daten eingehen, eine analoge Analyse nur mit der rechten Seite lieferte allerdings die gleiche statistische Aussage. Deutlich zu erkennen ist, dass die Höranstrengungskurven für einseitiges Hören zu höheren SNR nach rechts verschoben sind. Höranstrengungs-Ausgleichskurven, gemessen mit omnidirektionalem (schwarz) und direktionalem (grün) Programm, waren fast gleich. Die zugehörigen Boxplots der L1-, L7- und L13-Werte sind in Abb. 3 unten zu sehen. Der Friedman-Test ergab für die L1-Werte (χ^2^ = 20,9; *p* < 0,001), L7-Werte (χ^2^ = 17,5; *p* < 0,001) und L13-Werte (χ^2^ = 14,1; *p* = 0,003) eine signifikante Abhängigkeit von der Hörkondition (beidseitig/einseitig bzw. omnidirektional/direktional). Post-hoc-Wilcoxon-Rangsummentests ergaben hoch signifikante Differenzen zwischen beidseitigem und einseitigem Hören bei omnidirektionaler Einstellung (L1-Werte: *p* < 0,001; L7-Werte: *p* = 0,003; L13-Werte: *p* = 0,009) und bei direktionaler Einstellung (L1-Werte: *p* < 0,001; L7-Werte: *p* < 0,001; L13-Werte: *p* < 0,001). Die SNR-Differenz der Werte gleicher Höranstrengung (Mittelwert der Differenzen der L1-, L7-, und L13-Werte) betrug dabei omnidirektional 4,6 dB und direktional 7,4 dB.

Es zeigten sich allerdings keine signifikanten Unterschiede zwischen den Hörprogrammen (omnidirektional/direktional) bei einseitigem Hören (L1-Werte: *p* = 0,301; L7-Werte: *p* = 0,424; L13-Werte: *p* = 0,850).

## Diskussion

Diese Studie hat gezeigt, dass nur im Median eine numerisch geringere Höranstrengung bei Verwendung des direktionalen Programms, aber kein statistisch signifikanter Unterschied zwischen omnidirektionalem und direktionalem Programm nachzuweisen war, wenn die Probanden beidseitig gehört haben. Diese Erkenntnis widerspricht für die räumlichen Konditionen „S0°NCafe“, „S0°N180°“ und „S0°NRäumlich“ scheinbar der Erwartung einer deutlichen Verbesserung der Höranstrengung mit Richtmikrofon, welches andere Studien z. B. im Sprachverstehen [16, 20, 24] gefunden haben. Im direkten Vergleich mit [20] zeigte die hier vorliegende Studie mit VSB-Patienten z. B. in der Kondition „S0°NRäumlich“ eine Verschiebung der Höranstrengungskurve zu niedrigeren SNR im Median von 3,6 dB für mittlere und hohe Höranstrengungskategorien durch das Richtmikrofonprogramm, welche relativ gut zu dem in [20] für Normalhörende und bilateral Hörgeräteversorgte in einer ähnlichen akustischen Szene gefundenen Gewinn im Sprachverstehen von etwa 4 dB SNR passt. Für VSB-Träger hat [24] einen Gewinn von 2 bis 4,5 dB je nach Hörsituation gezeigt. Bezüglich der Höranstrengung zeigten [18] und [22] niedrigere Höranstrengung von bilateralen Hörgeräteträgern bei Vergleich von einem omnidirektionalen und direktionalen Programm und bei Vergleich von enger gegenüber weiter Richtwirkung [22] in ähnlicher Größenordnung. Die hier gefundene Verschiebung der Höranstrengungskurve zu niedrigeren SNR findet bei leicht negativen SNR statt. Solche Szenarien treten im Alltag selten auf [19], wenn sie aber auftreten, benötigen VSB-Träger besonders Hilfe durch die Signalverarbeitung.

Gründe dafür, dass unsere Studie keine statistische Signifikanz zwischen omnidirektionalem und direktionalem Programm gefunden hat, könnten sein, dass (1) das Messverfahren ACALES mit der Probandengruppe der VSB-Träger nicht sensitiv genug für diese Fragestellung ist, (2) die Signalverarbeitung im direktionalen Programm nicht effektiv genug ist, (3) das kontralaterale Ohr zu stark zur empfundenen Höranstrengung beiträgt.

Bezüglich des möglichen Grundes (1) bietet die mittlere interindividuelle Standardabweichung der individuellen Regressionskurven einen Anhaltspunkt. Diese beträgt für die Szenarien S0°N0° 3,6 dB, für S0°NCafe 4,8 dB, für S0°N180° 6,0 dB und für S0°NRäumlich: 5,3 dB, und ist damit ähnlich groß wie für Normalhörende [12]. Die intraindividuelle Standardabweichung, die wichtig für die Sensitivität des Tests ist, betrug in [12] 2,9 dB und sollte demnach ausreichen, um den erwarteten (und gefundenen) Gewinn durch die Signalverarbeitung von 3,6 dB nachzuweisen. Falls die intraindividuelle Standardabweichung für VSB-Träger nicht deutlich größer ist als für Normalhörende in [12], was aufgrund von fehlendem individuellen Test-Retest-Vergleich in der hier vorliegenden Studie leider nicht zweifelsfrei feststellbar ist, kann Grund (1), also die fehlende Sensitivität aufgrund großer intraindividueller Streuung, ausgeschlossen werden.

Die Literaturvergleichsstudien zur Untersuchung des Sprachverstehens [2, 16, 20] und [24] haben ähnliche Schalldruckpegel und SNR untersucht wie in dieser Studie. Auch die Differenz der Höranstrengungs-Regressionskurven zumindest in S0°NRäumlich ist ähnlich groß wie der Gewinn im Sprachverstehen in den Vergleichsstudien. Die Einregelzeit des Richtmikrofonprogramms im Audioprozessor ist zwar nicht bekannt, sollte aber aufgrund der hier verwendeten Signaldarbietung mit entsprechendem Störgeräusch-Vorlauf keine Rolle spielen. Daher ist insgesamt eine geringe Effektivität der Signalverarbeitung (Grund (2)) wenig wahrscheinlich.

Bezüglich des möglichen Grundes (3) ist zu beachten, dass das Richtmikrofonprogramm in dieser Studie nur für das implantierte Ohr verändert wurde, während in [2, 16, 18, 20] und [22] auf beiden Seiten der Programmwechsel simultan erfolgte. Die jeweiligen Einstellungen der Hörhilfen auf dem Gegenohr (HG, BB, VSB) wurden in dieser Studie nicht simultan verändert, und bei Normalhörigkeit kontralateral wäre dies natürlich auch nicht möglich gewesen. Dadurch, dass das Gegenohr in vielen Probanden eine mindestens ebenso wichtige Rolle spielt wie das implantierte Ohr, könnte der ansonsten nahezu SNR-unabhängige Effekt der Richtmikrofonwirkung deutlich reduziert worden sein. Zu ähnlichen Ergebnissen kommt die Studie von Kurz et al. (2020) [13] bei Vergleich des Effekts von omnidirektionaler und direktionaler Richtcharakteristik auf das Sprachverstehen von CI-Trägern mit kontralateral normalhörendem Ohr. Der Unterschied zwischen den Sprachverständlichkeitsschwellen mit und ohne Richtcharakteristik betrug dort im Mittel weniger als 1 dB und war statistisch nicht signifikant. Gegenüber der unversorgten Situation (CI abgeschaltet) ergaben sich allerdings zu beiden Mikrofoncharakteristiken hoch signifikante Unterschiede, was in der hier vorliegenden Studie nicht untersucht wurde. Um post hoc zu untersuchen, ob es einen Einfluss des Ohrs kontralateral zur VSB auf die Höranstrengung gibt, wurden die Probanden dieser Studie nachträglich gemäß PTA4_LL,kontralateral_ in zwei Gruppen eingeteilt, nämlich in eine kontralateral normalhörende Gruppe (Pat.-ID: 1, 7, 11, 12, 13, 14) und eine kontralateral schwerhörende Gruppe (Pat.-ID: 2, 3, 4, 8, 9, 10). Für S0°NRäumlich, bei dem im Probandenmittel die größten Abweichungen zwischen direktionalem und omnidirektionalem Programm gefunden wurden, zeigten sich Unterschiede zwischen den beiden Probandengruppen nur für das direktionale Programm für die L1-Werte (*p* < 0,001) und L7-Werte (*p* = 0,004). Hier zeigt die kontralateral normalhörende Gruppe niedrigere Höranstrengung als die kontralateral schwerhörende Gruppe, was einen Hinweis auf die generelle Beteiligung des Gegenohrs an der Höranstrengung liefert. Innerhalb jeder Probandengruppe zeigten sich allerdings keine signifikanten Unterschiede zwischen direktionalem und omnidirektionalem Programm (alle *p* > 0,05), sodass auch bei dieser zusätzlich durchgeführten Analyse die Nullhypothese von gleicher Höranstrengung in beiden Programmen nicht verworfen werden konnte.

Bei einseitigem Hören (Abb. 3) wurde in dieser Studie eine signifikant erhöhte Höranstrengung festgestellt, sowohl mit als auch ohne Richtmikrofonprogramm. Dies bestätigt die Wichtigkeit des Gegenohrs für die insgesamt wahrgenommene Höranstrengung und unterstreicht, was andere Studien schon mit VSB [23], aber auch mit anderen implantierten Hörsystemen, wie z. B. Cochleaimplantaten, gezeigt haben: Beidseitiges Hören ermöglicht Vorteile insbesondere in räumlichen Situationen. Dies wurde für die Lokalisationsfähigkeit [1], aber auch für das Sprachverstehen in räumlichen Situationen gezeigt (z. B. [2, 23, 25]). Alle Probanden dieser Studie verwenden im Alltag sowohl die VSB (Ø Tragezeit der VSB: 10,2 h/Tag) als auch die jeweiligen Hörhilfen auf dem Gegenohr. Die Probanden sind somit gewohnt, dass das Gegenohr im Alltag das Sprachverstehen verbessert und ergänzt. Die nicht vorhandenen signifikanten Unterschiede zwischen omnidirektionalem und direktionalem Programm sowohl bei einseitiger als auch beidseitiger Kondition unterstützen allerdings die Hypothese, dass das Gegenohr bezüglich der Auswirkungen der Audioprozessor-Signalverarbeitung im implantierten Ohr keine signifikante Rolle spielt.

Im Rahmen dieser Studie konnte gezeigt werden, dass das ACALES-Verfahren mit der Mehrzahl der VSB-Träger in einem Lautsprecherring, mit dem realistische räumliche Situationen simuliert werden, durchgeführt werden kann. Die Erfahrungen und Rückmeldungen aller Studienteilnehmer mit dem ACALES-Verfahren waren positiv hinsichtlich Studiendauer, Verständlichkeit und Durchführung der Studie und bestätigen bisher beschriebene Erfahrungen [12]. Die sechs unbeschrifteten Zwischenkategorien wurden trotz ausführlicher und wiederholter Einweisung der VSB-Träger selten verwendet.

In weiterführenden Studien könnten die unterschiedlichen Kopplungsmethoden des FMT bei ausreichend großer Anzahl an Studienteilnehmern auf signifikante Unterschiede verglichen werden, und es könnte untersucht werden, ob die Höranstrengung ohne die VSB-versorgte Seite (also reines kontralaterales Hören) unterschiedlich von der Höranstrengung bei beidseitigem Hören ist.

## Schlussfolgerung und Fazit für die Praxis

Diese Studie ermittelte die subjektive Höranstrengung mit dem ACALES-Verfahren von 15 VSB-Trägern in einem aus acht Lautsprechern bestehenden Kreis im reflexionsarmen Raum, wovon 12 Datensätze gemäß den Fragestellungen auswertbar waren. Vier verschiedene akustische Szenarios wurden mit unterschiedlichen Störgeräuschen und mit dargebotenen Sätzen des Oldenburger Satztests als Zielsignal verwendet.

Folgende Erkenntnisse wurden dabei gewonnen:

- (1) Die Verwendung des Richtmikrofonprogramms der VSB führte bei den Probanden nicht zu der ursprünglich erwarteten signifikanten Verbesserung der Höranstrengung. Allerdings gab es in den Konditionen mit räumlich verteilten Störschallquellen, insbesondere bei negativen Signal-Rausch-Verhältnissen insgesamt eine im Median geringe Verbesserung der Höranstrengung. Die Differenz der Höranstrengungs-Regressionskurven lag hierbei in Einklang mit Literaturwerten zu Verbesserungen im Sprachverstehen mit Richtmikrofonie.
- (2) Beidseitiges Hören ermöglichte für VSB-Träger bei allen hier untersuchten Konditionen eine signifikante Verbesserung der Höranstrengung gegenüber dem Verschluss des Ohrs und einseitigem Hören nur mit der VSB. Patienten sollten daher ermutigt werden, immer möglichst mit beiden Ohren bestmöglich versorgt zu hören.
